# qPCR-Based Molecular Detection of *Trichophyton indotineae* by Targeting Divergent Sequences

**DOI:** 10.1007/s11046-025-00939-5

**Published:** 2025-04-03

**Authors:** Engin Kaplan, Oğuzhan Bingöl, Hazal Kandemir, Ayşe Sultan Karakoyun, Murat Durdu, Macit Ilkit

**Affiliations:** 1 Department of Pharmaceutical Microbiology, Faculty of Pharmacy, Istanbul University-Cerrahpaşa, Istanbul, Türkiye; 2 Division of Mycology, Department of Microbiology, Faculty of Medicine, Çukurova University, Adana, Türkiye; 3 Westerdijk Fungal Biodiversity Institute, Utrecht, The Netherlands; 4 Present Address: Department of Environmental Biology, Institute of Environmental Sciences (CML), Leiden University, Leiden, The Netherlands; 5 Department of Dermatology, Faculty of Medicine, Adana Dr. Turgut Noyan Application and Research Center, Başkent University, Adana, Türkiye

**Keywords:** Dermatophyte, Diagnostics, Identification, Real-time PCR, Drug resistance

## Abstract

*Trichophyton indotineae*, formerly known as *T*. *mentagrophytes* internal transcribed spacer (ITS) genotype VIII, has been recognized over the last decade due to its high virulence and resistance to treatment. Its accurate identification in routine mycology laboratories remains challenging, as it shares phenotypic traits and substantial rDNA ITS similarity with *T*. *mentagrophytes* and *T*. *interdigitale*. This study aimed to identify more divergent and stable sequences via whole-genome comparisons between *T*. *indotineae* and *T*. *interdigitale* to facilitate highly specific targeting of *T*. *indotineae* using a validated quantitative polymerase chain reaction (qPCR)-based method. Our whole-genome comparison revealed at least 22 unique sequences of *T*. *indotineae* compared to *T*. *interdigitale* and revealed the divergence of the former from the reference genomes of other *Trichophyton* species. Among these, a 499 bp segment was identified as the most genetically distinct sequence within the *T*. *indotineae* genome. Seventy-three dermatophyte strains [*T*. *indotineae* (*n* = 66), non-*T*. *indotineae* (*n* = 7)], were tested using our qPCR assay targeting the above-mentioned stable 499-bp region. Regarding analytical performance, our *T*. *indotineae*-specific qPCR assay exhibited high sensitivity (93.3%) and specificity (100%), with a detection limit of ~ 15 genomic copies. Our approach has the potential to establish highly sensitive and specific qPCR assays without relying on specialized assay designs for single nucleotide polymorphisms in the ITS or other loci. This approach offers a practical solution for updating molecular diagnostics, particularly for novel taxa such as *T*. *indotineae*, for which limited gene data are available in public databases.

**Supplementary Information:**

The online version contains supplementary material available at 10.1007/s11046-025-00939-5.

## Introduction

Dermatophytes are common pathogens of the skin, hair, and nails that infect approximately one-quarter of the world’s population [1]. *Trichophyton*, including *T*. *rubrum* and *T*. *mentagrophytes* species complexes, is the most common genus among dermatophytes. These complexes cause severe, antifungal-resistant dermatophytosis and have significant public health implications [2]. *T*. *indotineae* is a novel species whose nomenclature remains controversial among researchers [3]. It was formerly known as *T*. *mentagrophytes* ITS genotype VIII. Over the last decade, outbreaks of *T*. *indotineae* have raised concerns because of its high virulence and the difficulty in treating such infections [4, 5].

*Trichophyton indotineae* is an important causative agent of ținea corporis and tinea cruris, presenting in inflammatory, chronic and relapsing forms across all ages and sexes [6]. The risks posed by *Trichophyton* infections, including those by *T*. *indotineae*, are exacerbated by point mutations in the squalene epoxidase (*SQLE*) gene as the predominant source of resistance against terbinafine, an important drug for first-line therapy against such infections [7–9]. Additionally, infections caused by *T*. *indotineae* are increasing worldwide, including across Asia, the Middle East, Europe, and the USA [10, 11]. To counter these challenges and achieve effective infection control, rapid and accurate diagnosis of *T*. *indotineae* infections is urgently required.

The identification of *T*. *indotineae* in routine mycology laboratories is problematic because of its phenotypic and high rDNA internal transcribed spacer (ITS) similarities (99%) with *T*. *mentagrophytes* and *T*. *interdigitale* [12, 13]. Although public databases (e.g., NCBI GenBank and Assembly) contain a remarkable number of raw whole-genome sequences of *T*. *indotineae*, the available nucleotide sequences are inadequate and mostly contain partial rDNA regions and a few sequences of *ERG1* and *ERG11* (https://www.ncbi.nlm.nih.gov/nuccore, accessed on: 15/12/2024).

The alarming increase in *T*. *indotineae* infections globally highlights the need for developing novel tools for rapid and inexpensive diagnosis. However, to more accurately identify *T*. *indotineae*, there is an urgent need to obtain more discriminatory and stable coding and non-coding sequences. In this study, we aimed to compare the homologs of several known protein-coding loci and reference whole-genome sequences of *T*. *indotineae* with those of *T*. *interdigitale* and other members of the *T*. *mentagrophytes* species complex. The goal was to identify more divergent and stable sequences to enable highly discriminatory targeting of *T*. *indotineae* through a quantitative polymerase chain reaction (qPCR)-based assay.

## Materials and Methods

### Isolates

We analyzed a total of 73 dermatophyte strains [*T*. *indotineae* (*n* = 66), *T*. *rubrum* (*n* = 5), *T*. *tonsurans/equinum* (*n* = 1), and *Microsporum* sp. (*n* = 1)] from our laboratory collection which were previously recovered from patients with chronic and treatment-resistant dermatophytosis in Türkiye. For each strain, species identification was recently confirmed by our group based on multilocus sequence typing (MLST) of the ITS, LSU, partial *BTUB*, and *Tef1-*α loci (Durdu M, et al., 2025. In preparation). The anatomical and geographical origins of these strains are listed in Table 1.

**Table 1:** Isolates analyzed in this study

Species	Isolate No.	Source	Geography	qPCR
*T*.* indotineae*	MI-1	LE	Istanbul	P
*T*. *indotineae*	MI-3	UE, AT, F	Istanbul	P
*T*.* indotineae*	MI-5	LE	Istanbul	P
*T*.* indotineae*	MI-6	LE, F	Istanbul	P
*T*.* indotineae*	MI-7	LE, AT	Istanbul	P
*T*. *indotineae*	MI-9	LE, IT	Istanbul	P
*T*.* indotineae*	MI-11	AT, IR, F	Istanbul	P
*T*.* indotineae*	MI-15	LE, UE, IR	Istanbul	P
*T*. *indotineae*	MI-16	LE, UE, AT, IT	Istanbul	P
*T*.* indotineae*	MI-17	LE, IR	Istanbul	P
*T*.* indotineae*	MI-18	LE, IR	Istanbul	P
*T*. *indotineae*	MI-20	UE, AT, IT	Istanbul	P
*T. indotineae*	MI-21	LE, AT, IR	Istanbul	P
*T*.* indotineae*	MI-22	LE, UE, AT, IT, IR	Istanbul	P
*T*. *indotineae*	MI-23	LE, UE, AT, IR	Istanbul	P
*T*. *indotineae*	MI-25	AT, IR	Istanbul	P
*T*.* indotineae*	MI-26	UE	Istanbul	P
*T*.* indotineae*	MI-27	UE, AT	Istanbul	N
*T*.* indotineae*	MI-1128	UE, IR	Adana	P
*T*.* indotineae*	MI-1177	AT, IT, F, IR, UE	Adana	P
*T*.* indotineae*	MI-1179	IT, UE, LE	Adana	P
*T*.* indotineae*	MI-1182	UE, LE, AT, IT, IR	Adana	P
*T*.* indotineae*	MI-1194	LE, AT, IR	Kahramanmaraş	P
*T*.* indotineae*	MI-1195	IT, IR, F	Istanbul	P
*T*.* indotineae*	MI-1196	UE, LE, AT, F	Muğla	P
*T*.* indotineae*	MI-1197	AT, IT, UE, IR	Adana	P
*T*. *indotineae*	MI-1199	LE	Adana	P
*T*.* indotineae*	MI-1204	IR	Sanlıurfa	P
*T*.* indotineae*	MI-1206	LE, AT, IR	Adana	P
*T*. *indotineae*	MI-1207	IT, IR, LE	Adana	P
*T*.* indotineae*	MI-1208	LE, UE	Adana	N
*T*.* indotineae*	MI-1210	AT, IR	Muğla	P
*T*.* indotineae*	MI-1211	IR	Adana	P
*T*.* indotineae*	MI-1212	UE, LE, IR	Adana	P
*T*. *indotineae*	MI-1213	LE, UE, AT, IR, F	Adana	P
*T*. *indotineae*	MI-1215	UE, IR	Adana	P
*T*. *indotineae*	MI-1219	UE, LE, AT, IT, IR	Adana	P
*T*.* indotineae*	MI-1221	LE, IR	Adana	P
*T*.* indotineae*	MI-1225	AT, IR	Adana	P
*T*.* indotineae*	MI-1227	IR	Adana	P
*T*.* indotineae*	MI-1230	LE, AT, IR	Muğla	P
*T. indotineae*	MI-1231	LE, AT, F	Muğla	N
*T*.* indotineae*	MI-1232	IR	Adana	P
*T*.* indotineae*	MI-1234	LE, AT, IR	Muğla	P
*T*.* indotineae*	MI-1236	F, AT, LE	Muğla	P
*T*. *indotineae*	MI-1244	AT, IT, IR	Muğla	P
*T*.* indotineae*	MI-1252	LE, IT	Kahramanmaraş	P
*T*.* indotineae*	MI-1253	LE, AT, IT	Kahramanmaraş	P
*T*.* indotineae*	MI-1254	LE, AT, IT, IR	Kahramanmaraş	P
*T*.* indotineae*	MI-1255	LE, UE, AT, IT, IR	Kahramanmaraş	P
*T*. *indotineae*	MI-1257	LE, UE, IR	Kahramanmaraş	P
*T*.* indotineae*	MI-1259	LE	Kahramanmaraş	P
*T*. *indotineae*	MI-1261	LE, UE, AT, IT, IR	Kocaeli	P
*T*.* indotineae*	MI-1262	AT, IT	Kahramanmaraş	P
*T*. *indotineae*	MI-1263	LE, UE, AT, IT, IR	Adana	P
*T*.* indotineae*	MI-1264	LE, UE, IR	Adana	P
*T*.* indotineae*	MI-1265	UE, AT	Adana	P
*T*. *indotineae*	MI-1271	LE, IR	Adana	P
*T*.* indotineae*	MI-1272	LE, UE, AT, IT, IR	Adana	N
*T*.* indotineae*	MI-1273	UE, AT, F, IR	Adana	P
*T*. *indotineae*	MI-1275	IR	Adana	P
*T*.* indotineae*	MI-1276	IR, F	Kahramanmaraş	P
*T*.* indotineae*	MI-1281	LE, AT, IT, IR	Bartın	P
*T*.* indotineae*	MI-1286	IR	Adana	P
*T*.* indotineae*	MI-1287	LE, UE, AT, IT, IR, F	Adana	P
*T*.* indotineae*	MI-1289	LE, UE, AT, IT, IR, F	Adana	P
*T. rubrum*	MI-12	LE	Istanbul	N
*T. rubrum*	MI-24	LE, AT, IR	Istanbul	N
*T. rubrum*	MI-1185	LE, IR	Adana	N
*T*.* rubrum*	MI-1266	LE, UE, AT, IR	Adana	N
*T*.* rubrum*	MI-4	LE, UE, AT	Istanbul	N
*T*. *tonsurans/equinum*	MI-1198	AT, IT, IR	Adana	N
*Microsporum* sp.	MI-19	IR	Istanbul	N

UE, Upper extremity; LE, Lower extremity; AT, Anterior aspect of the trunk; IT, Inferior aspect of the trunk; IR, Inguinal region; P, positive; N, negative; MI, Macit Ilkit Working Collection

*Trichophyton indotineae* CBS 149165 was used in this study. Non-*T*. *indotineae* reference strains, including *T*. *interdigitale* (DSMZ 4167), *T*. *tonsurans* (DMSZ 12285), *Microsporum canis* (DMSZ 10708), and *Nannizzia gypsea* (DSMZ 3824), were obtained from the culture collection of DSMZ (Leibniz Institute DSMZ-German Collection of Microorganisms and Cell Cultures, Brunswick, Germany). All strains were incubated on Sabouraud glucose agar (Merck, Darmstadt, Germany) plates at 28 °C for 10 days before analysis.

### DNA Extraction

Genomic DNA was extracted using the cetrimonium bromide protocol [14]. DNA quality was determined using a BioTek Synergy H1 Multimode Reader spectrophotometer (Agilent Technologies, Santa Clara, CA, USA).

### Individual Genes and Whole-Genome Comparisons

We performed BLAST analyses in NCBI GenBank for inter-strain comparison of the ITS region and several protein-coding genes including *ACT*, *BTB*, *CaM*, *CHS1*, *HSP70*, *RPB1-2*, *Tef1-*α, and *TUB2*, which are commonly used in MLST of dermatophytes [15, 16]. The reference whole genomes, namely, those of *T*. *indotineae* (GCA_023065905.1) and *T*. *interdigitale* (GCA_037576225.1), were used together with eight additional whole genomes of *T*. *indotineae* (GCA_023065865.1, GCA_023065795.1, GCA_023065885.1, GCA_032157395.1, GCA_023065845.1, GCA_023065815.1, GCA_032157385.1, and GCA_032157405) in the NCBI Assembly database (https://www.ncbi.nlm.nih.gov/) to compare the above-mentioned loci for possible diversity. In addition to analyzing the multiple loci, we compared the reference whole genomes of *T*. *indotineae* and *T*. *interdigitale* to screen for any possible divergent and stable genome regions that could improve the ability to discriminate *T*. *indotineae*.

MAFFT [17] and Mauve [18, 19] multiple sequence alignment tools were used as plugins in the trial version of Geneious Prime v.2025.0.3 (Dotmatics, Boston, MA, USA) to perform whole-genome sequence alignment and to visualize genome comparisons. The distinct or most divergent sequences were identified from the aligned contigs and subsequently verified through NCBI BLAST analysis against *T*. *interdigitale* (GCA_037576225.1) as well as several closely related species, including *T*. *mentagrophytes* (GCA_003664465.1), *T*. *benhamiae* (GCA_000151125.2), *T*. *tonsurans* (GCA_000151455.1), *T*. *schoenleinii* (GCA_018357685.1), and *T*. *rubrum* (GCA_000151425.1). Owing to the lack of available RefSeq data for *T*. *indotineae* and *T*. *interdigitale* in public repositories, the RefSeq data for *T*. *benhamiae* were used to conduct an NCBI BLAST analysis to determine the possible coding features.

Interspecies stability and any possible divergence of the obtained sequence regions were predicted via in silico searches through the above-mentioned eight non-reference whole-genome sequences of *T*. *indotineae* and via temperature variability through melting curve analysis.

### qPCR Assay Design

A qPCR assay targeted the most divergent and stable sequences determined from a whole-genome comparison between *T*. *indotineae* and *T*. *interdigitale*. For primer design, one of the unique or most divergent genomic regions of *T*. *indotineae* was extracted in silico from the reference whole-genome and analyzed using MEGA X software [20]. The optimal oligonucleotide-binding regions were checked for their thermodynamic features using Oligo Analyzer v3.1 (https://eu.idtdna.com/calc/analyzer) [21], and the specificity of the oligonucleotides was predicted using Primer-Blast (www.ncbi.nlm.nih.gov/tools/primer-blast). The designed oligonucleotides were synthesized by Probesynthesis Biotechnology (Istanbul, Türkiye).

The qPCR assay was performed with SYBR Green in a total reaction volume of 25 μL, consisting of 12.5 μL of BlasTaq™ 2X qPCR Master Mix (ABM, Vancouver, Canada), 0.5 μL of each primer (10 μM), and 11.5 μL of PCR-grade water via the CFX96 Touch Real-Time PCR Detection System (Bio-Rad, Hercules, CA, USA). The PCR conditions included an initial denaturation step at 95 °C for 3 min, followed by 40 cycles of 95 °C for 30 s, 55 °C for 30 s, and 72 °C for 30 s. Meanwhile, melting curve analysis was performed under the following conditions: 95 °C for 15 s, 65 °C for 1 min, and 95 °C for 15 s.

### qPCR Assay Validation

Validation was performed to evaluate the performance of qPCR assays. Seventy-three isolates were tested to determine the sensitivity of the assay. To determine the specificity, reference strains of *T*. *interdigitale* (DSMZ 4167), *Microsporum canis* (DMSZ 10708), *N*. *gypsea* (DSMZ 3824), and *T*. *tonsurans* (DMSZ 12285) (Table 1) were included. In addition to the clinical isolates and reference fungal strains, human DNA from cell culture extracts (keratinocytes, HaCaT) was included in the qPCR assay to screen for possible cross-reactions. The sensitivity and specificity of the assay were calculated according to the methods described by Blakely and Salmond [22].

**Fig. 1:**
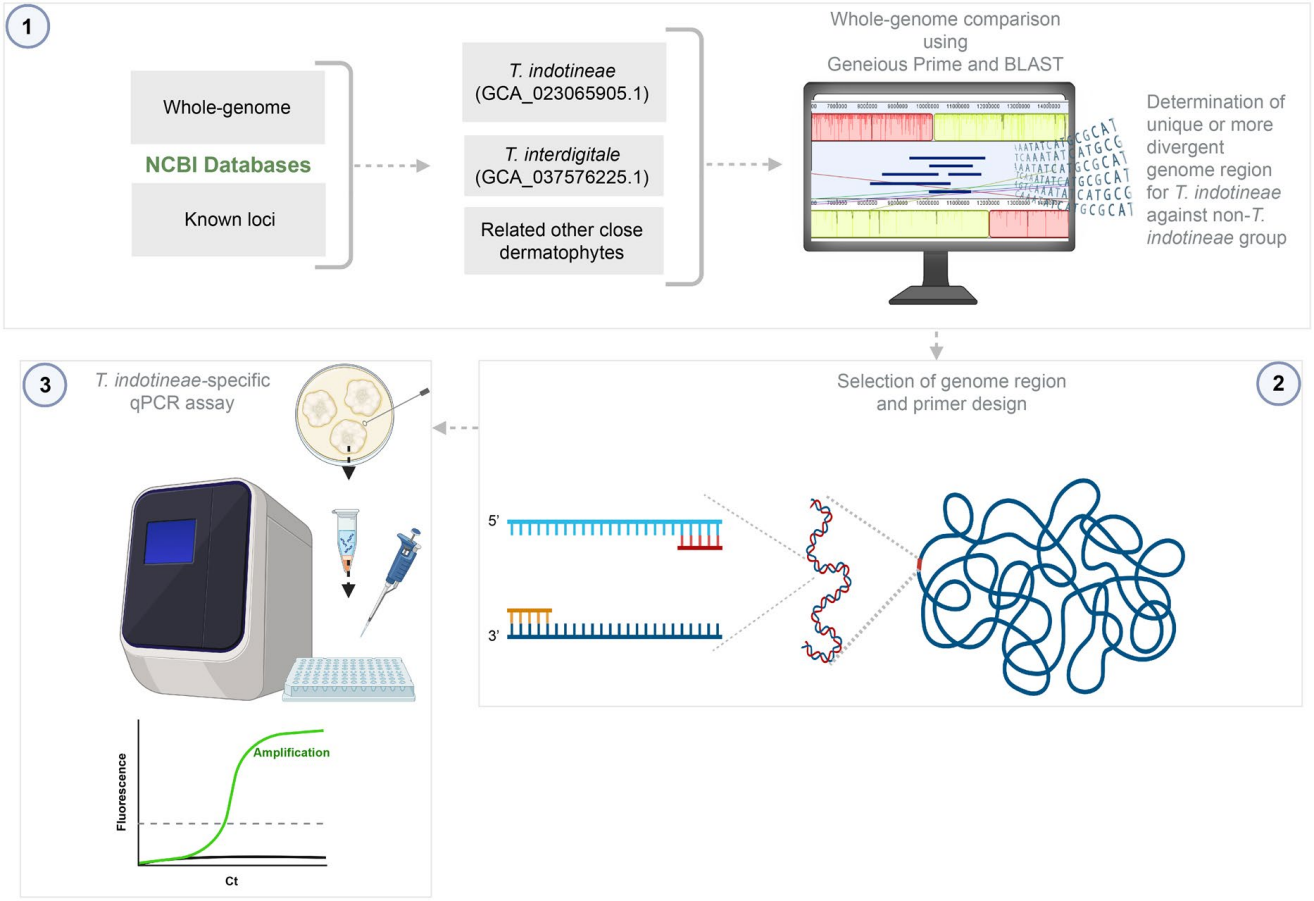
Overview of the study workflow. (1) Identification of unique sequences specific to *Trichophyton indotineae* through comparative genomic analysis using Geneious Prime and NCBI Blast, (2) design and optimization of specific primer sets for *T*. *indotineae*, and (3) development of a quantitative polymerase chain reaction (qPCR) assay for detection of *T*. *indotineae* from other dermatophytes (the figure was created using BioRender [BioRender.com])

Genomic DNA from *T*. *indotineae* (CBS 149165) was tested using analytical parameters such as slope, efficiency, linearity, repeatability, and detection limit, in accordance with the Minimum Information for Publication of Quantitative Real-Time PCR Experiments guidelines [23]. Values were calculated using Bio-Rad CFX Maestro v1.1. The DNA copy number was calculated using a DNA Copy Number Calculator (Thermo Scientific Webtools), with a genome size of 22.3 Mb (NCBI Assembly: GCA_023065905.1). The repeatability and detection limit of the assay were assessed by testing the DNA amounts equivalent to 1,000 copies and the minimum detectable theoretical copy number, respectively, in five replicates under the same reaction and thermal cycler conditions. The overall workflow of the experimental setup of the study is illustrated in Fig. 1.

## Results

### Individual Gene and Whole-Genome Comparisons

Due to the limited availability of *T*. *indotineae* gene sequences in public databases, in addition to the generation of raw whole-genome sequences, we performed an in-silico analysis to identify and compare the homologous loci of genes commonly targeted for differentiating dermatophytes, including partial or complete sequences of *ACT*, *BTB*, *CaM*, *CHS1*, *HSP70*, *RPB1-2*, *Tef1-*α, and *TUB2.* Our analysis revealed a high degree of similarity for all loci, ranging from 99.4% to 100% (Table S1), with most sequences differing between *T*. *indotineae* and *T*. *interdigitale* by only one or two bases. No differences were found in the sequences of these multiple loci when compared between the reference and other whole-genome sequences of *T*. *indotineae* available in the NCBI Assembly database.

Given the high similarity of the known loci between *T*. *indotineae* and *T*. *interdigitale*, we compared their whole-genome reference sequences. The compared genomes comprise 22.3 Mb for *T*. *indotineae* (GCA_023065905.1) and 22.2 Mb for *T*. *interdigitale* (GCA_037576225.1). In this comparison, we screened unique sequences greater than 150 bp in length to facilitate more effective qPCR targeting. Our analysis identified at least 22 unique sequences of this type with lengths of up to 1,175 bp. The contig JAJVHL010000001.1 of *T*. *indotineae* (GCA_023065905.1) contained 59% (13 out of 22) of the identified unique sequences. The remaining unique sequences were extracted from contigs JAJVHL010000002.1 and JAJVHL010000003.1 of the reference whole genome. The unique sequences identified were further validated through BLAST analysis, confirming their dissimilarity from the reference genome sequence of *T*. *interdigitale* (GCA_037576225.1). GenBank accession numbers for the unique sequences of *T*. *indotineae* are listed in Table S1.

The extracted sequences of *T*. *indotineae* that were not found in *T*. *interdigitale* were also analyzed against the reference genomes of *T*. *benhamiae*, *T*. *mentagrophytes*, *T*. *rubrum*,* T*. *schoenleinii*, and *T*. *tonsurans*. BLAST analysis revealed that some of the identified sequences of *T*. *indotineae* were found in one or more species. However, these sequences were still particularly divergent, making them more suitable for targeting than other loci commonly used for differentiating dermatophytes, including the ITS. In comparison with the whole-genome mRNA sequences of *T. benhamiae*, many of the sequences identified in *T. indotineae *were found to share part or all of their coding sequences (Table S1). Among the identified sequences, we selected a 499 bp region as the most divergent, with the genome position of JAJVHL010000003.1:1,936,972–1,937,471 in *T. indotineae* (GCA_023065905.1), which appears to be part of a promoter region. The selected region showed no similarity to the sequences of *T*. *interdigitale*, *T*. *mentagrophytes*, *T*. *rubrum*, and *T*. *tonsurans*. However, it showed sufficient dissimilarity to *T*. *benhamiae* and *T*. *schoenleinii*, with query coverage versus percentage identity values of 98% vs. 77% and 100% vs. 88%, respectively.

### qPCR Assay Design, Analysis, and Validation

As our selected target sequence showed sufficient dissimilarity to those of *T*. *mentagrophytes*, *T*. *interdigitale*, *T*. *tonsurans*, or *T*. *rubrum*, we focused on aligning homologous sequences from *T*. *benhamiae* and *T*. *schoenleinii* to identify regions that diverged between them to design a primer set for our qPCR assay. In silico specificity analysis using Primer-BLAST (www.ncbi.nlm.nih.gov/tools/primer-blast) predicted that the primers would not cross-react with any of the dermatophyte or human genome. Therefore, the qPCR assay was designed with the primer set Tindo_F (5′-ATAGAAGTTATTCTAAAAGATATCGA-3′) and Tindo_R (5′-TGCGATATTAGATATTAGGAGCTCTA-3′), which generates a 445 bp amplicon.

**Fig. 2:**
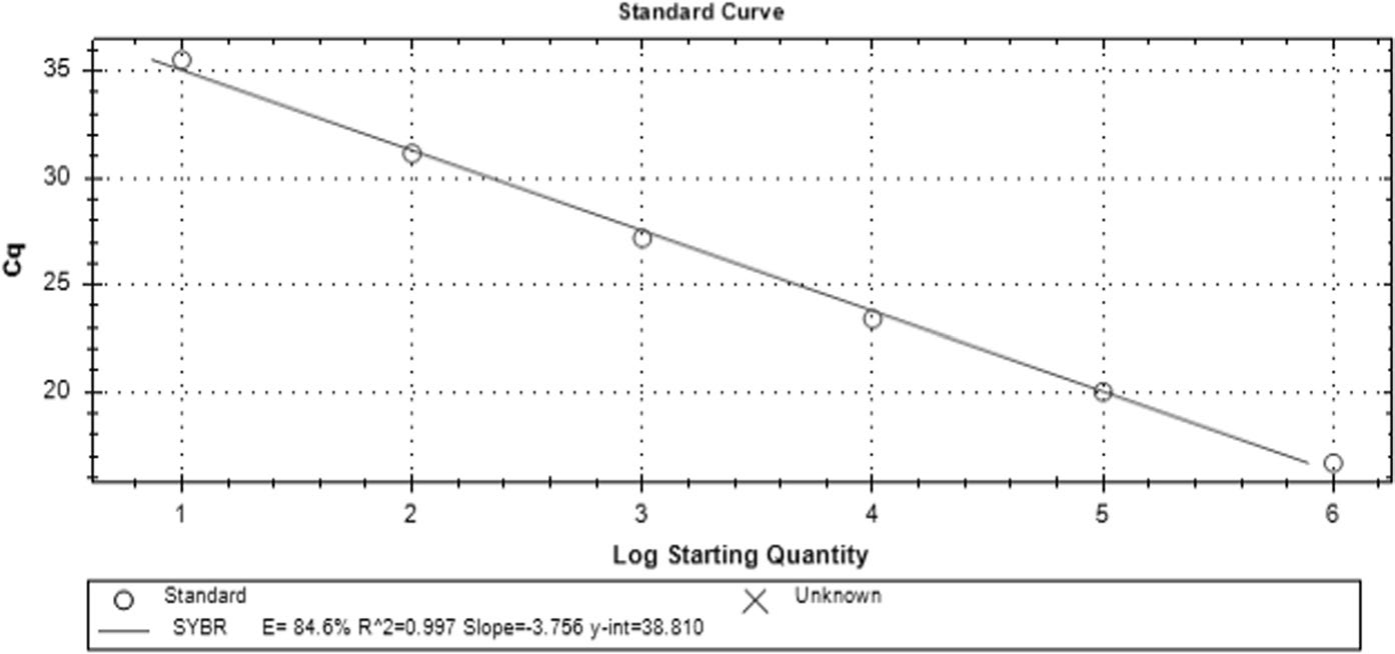
Standard curve of the *T. indotineae*-specific qPCR assay, which targets gDNA at concentrations ranging from 3.5 × 10^1^ to 3.5 × 10^–4^ ng/μL (approximately 150,000 to 15 theoretical copies)

Our *T. indotineae* qPCR assay successfully identified the reference *T. indotineae* strain (CBS 149165) and showed no cross-reactivity with the reference non-*T. indotineae* dermatophytes, including *T. interdigitale* (DSMZ 4167), *T. tonsurans* (DSMZ 12285), *Microsporum canis* (DSMZ 10708), and *N*. *gypsea* (DSMZ 3824). The sensitivity and specificity of the *T*. *indotineae* qPCR assay were 93.3% and 100%, respectively, with no cross-reactivity against the non-*T. indotineae* dermatophytes included in this study or against the human genome. The melting temperature of positive qPCR assays was consistently 74 °C (± 0.5) across all positive isolates. Agarose gel electrophoresis confirmed the stability of the genomic region in terms of length, as the amplicon lengths were identical in all positive qPCR assays. The genome of *T*. *indotineae* is 22.3 Mb in size, corresponding to approximately 41,545 theoretical copies per ng of DNA. The lower theoretical detection limit was approximately 15 genome copies, with Ct values ranging from 35 to 38. The assay demonstrated a coefficient of determination (R^2^) of 0.997, a slope of −3.756, and an efficiency (E) of 84.6% (Fig. 2). The relative standard deviation for repeatability was 1.5% for 1,000 genome copies and 2.5% for the theoretical detection limit of 15 copies (Fig. S1).

## Discussion

In this study, we analyzed and compared various homologs of commonly targeted loci for the molecular identification of dermatophytes, focusing on the reference genomes of *T*. *indotineae* and *T*. *interdigitale*. Considering the high similarity of multiple known loci between these two species, which varies between 99.4% and 100%, we compared their reference genomes to screen for unique or particularly diverse sequences. This analysis revealed at least 22 unique genomic regions in *T*. *indotineae* ranging in length from 150 to 1,175 bp. BLAST analysis of these unique sequences against *T*. *benhamiae*, *T*. *interdigitale*, *T*. *rubrum*, *T*. *schoenleinii*, and *T*. *tonsurans* identified a specific genomic region of *T*. *indotineae* located on JAJVHL010000003.1:1,936,972–1,937,471 (GCA_023065905.1), that presented the greatest divergence between the species. This region was selected and targeted in our qPCR assay using the newly designed primers Tindo-F and Tindo-R, which are specific to *T*. *indotineae*. The sensitivity and specificity of the qPCR assay were 93.3% and 100%, respectively. Validation tests confirmed the applicability of the assay to specifically target *T*. *indotineae*.

Although this issue remains controversial, in clinical practice, *T*. *indotineae* is considered a novel taxon rather than a distinct clone and is referred to as *T*. *mentagrophytes* ITS genotype VIII within the *T*. *mentagrophytes* complex, mainly owing to its resistance to multiple antifungal drugs and high virulence [3, 11, 12, 24]. Based on ITS sequences, *T. indotineae* differs from *T. mentagrophytes* and *T. interdigitale* by a single nucleotide polymorphism (SNP), while no variation was observed among *T. indotineae*, *T. benhamiae*, and *T. rubrum* at the same position. Although the *HMG* gene was highly variable in *T*. *indotineae* / *T*. *interdigitale* / *T*. *mentagrophytes*, it is not a sufficiently stable region across species because of its association with the mating type mechanism [13].

In addition to the ITS region, our comparison of the full reference genomes of *T*. *indotineae* and *T*. *interdigitale* revealed that commonly targeted loci in multilocus analyses, including *Tef1-*α, *ACT*, *TUB2*, *CaM*, *RPB1-2*, *HSP70*, *CHS1*, and *BTB*, had remarkably high sequence similarity (varying between 99.4% and 100%) between the two species. Additionally, although two or more distinct regions may provide sufficient variants for multilocus analysis, such an approach is impractical as a rapid and cost-effective diagnostic tool for identifying this group of fungi.

To improve the differential diagnosis of *T*. *indotineae* from the *T*. *mentagrophytes* complex and other dermatophytes, several molecular tools, primarily those based on qPCR and matrix-assisted laser desorption/ionization time-of-flight mass spectrometry (MALDI-TOF MS), have been employed. Baron et al. [25] were the first to develop ITS-based SNP qPCR for the diagnosis of *T*. *indotineae* isolates from both fungal cultures and direct clinical samples with 100% specificity and sensitivity. Rouhi et al. [26] developed a qPCR assay targeting two non-ITS regions and tested 397 *T*. *indotineae* and non-*T*. *indotineae* strains that were previously evaluated by Batvandi et al. [27] using PCR-restriction fragment length polymorphism (PCR–RFLP) analysis, focusing on the DNA topoisomerase IIα (*TOP2*) gene. Additionally, Normand et al. [28] used MALDI-TOF MS to distinguish *T*. *indotineae* from *T*. *interdigitale/T*. *mentagrophytes*, achieving an accuracy of up to 97.4% across various culture conditions. De Paepe et al. [29] combined their in-house BCCM/IHEM database with an online MSI-2 tool developed by Normand et al. [28] to identify 100% of strains as *T*. *indotineae*. Furthermore, Tang et al. [24] compared qPCR, loop-mediated isothermal amplification (LAMP), MALDI-TOF MS, and the commercially available DermaGenius 2.0, which targets ITS, to distinguish species in the *T*. *mentagrophytes* complex. They reported insufficient discrimination using qPCR and WarmStart colorimetric RT-LAMP techniques. DermaGenius 2.0, which lacks a specific probe targeting *T*. *indotineae*, demonstrated the capacity to discriminate diagnostic capability only between *T*. *interdigitale* and *T*. *mentagrophytes* directly from 49 skin scrapings and hair samples. In contrast, the authors distinguished 96.9% of *T*. *indotineae* isolates from other closely related species using MALDI-TOF MS.

While several studies have reported the design of differential diagnostic assays targeting ITSs [25], others have reported that ITS sequencing is inadequate for distinguishing *T*. *indotineae* from closely related species [24, 26, 27]. This highlights the need to identify more discriminative and stable genomic regions for more accurate identification and differentiation of *T*. *indotineae*. In this study, we compared the whole-genome of this species to address this challenge. This approach is an effective and promising strategy for advancing molecular diagnostics, particularly in response to the evolving fungal taxonomy. Moreover, it is well suited for identifying novel taxa, such as *T*. *indotineae*, for which sufficient molecular data are often lacking in public databases.

Although the sensitivity of our qPCR assay in clinical samples clearly depends on the efficiency of the DNA isolation method employed, it offers a rapid (2-h/test) and cost-effective (up to $3/test) solution in comparison to the existing limited number of dermatophyte-specific commercial diagnostic kits (e.g., DermaGenius 2.0 kit, ~ $16/test) for the routine screening of *T*. *indotineae*, making it particularly valuable in developing countries. However, our test is a single-plex assay, capable of identifying only* T*. *indotineae*, unlike the multiplex kits offered by DermaGenius. It may also be necessary to further validate our assay using additional non-*T*. *indotineae* isolates. These aspects can also be considered as disadvantages of the technique. The main limitation of this study was the relatively small number of non-*T*. *indotineae* strains that were analyzed.

## Conclusions

In this study, we developed a qPCR assay to address the urgent need for improved diagnosis of *T*. *indotineae* infections. This technique provides a validated, highly sensitive, and specific diagnostic approach without requiring specialized qPCR designs targeting SNPs in the ITS or other loci. The reliable performance and speed of qPCR, along with its low-cost for identifying the emerging treatment-resistant pathogen *T*. *indotineae*, make it a valuable technique. This method could aid in treatment decision-making, predict response to therapy, facilitate follow-up, and improve patient outcomes. Future studies should focus on evaluating this assay in routine clinical settings using a diverse range of clinical samples.

## Supplementary Information

Below is the link to the electronic supplementary material.

Supplementary file1 (DOCX 42 kb)
