# Supplementary material for: qPCR-Based Molecular Detection of Trichophyton indotineae by Targeting Divergent Sequences
Source: Mycopathologia. 2025 Apr 3;190(2):32. doi: 10.1007/s11046-025-00939-5 (PMC11968514; doi:10.1007/s11046-025-00939-5)
Supplement: Supplementary file 1 — Supplementary file1 (DOCX 42 kb) [file 11046_2025_939_MOESM1_ESM.docx]

**Table S1** Data obtained from whole-genome comparison and BLAST analysis between *T*. *indotineae* and *T*. *interdigitale*/*T*. *benhamiae* (RefSeq)

| \| **Location (***T. indotineae*: GCA_023065905.1) \| **Gene/Protein name**^‡^ \| **Similarity (%)^#^** \| \| --- \| --- \| --- \| \| NR_173767.1 \| ITS \| 99.49 \| \| JAJVHL010000001.1:c7457134-7455215 \| TEF1a \| 99.53 \| \| JAJVHL010000001.1:1788802 to 1789872 \| Actin \| 99.95 \| \| JAJVHL010000001.1: 3624434 to 3624940 \| TUB2 \| 99.41 \| \| JAJVHL010000001.1: 2379056 to 2379720 \| CaM \| 99.55 \| \| JAJVHL010000003.1: 3693757 to 3696933 \| RPB1 \| 99.59 \| \| JAJVHL010000002.1: 2287342 to 2290668 \| RPB2 \| 99.79 \| \| JAJVHL010000002.1:1239187 to 1239789 \| CHS1 \| 100.00 \| \| JAJVHL010000001.1: 2116182 to 2117097 \| HSP70 \| 99.45 \| \| JAJVHL010000002.1: 3146933 to 3151864 \| BTB \| 99.72 \| \| **Location (***T. indotineae*: GCA_023065905.1) \| **Gene/Protein name**^§^ \| **Similarity (%)^#^** \| \| JAJVHL010000001.1:879003-879161 \| ND \| NS \| \| JAJVHL010000001.1:380701-380887 \| ARB_03826 \| NS \| \| JAJVHL010000001.1:1035316-1035508 \| ARB_00207 \| NS \| \| JAJVHL010000001.1:258263-258472 \| ARB_03866 \| NS \| \| JAJVHL010000001.1:2624311-2624536 \| ARB_04407 \| NS \| \| JAJVHL010000001.1:5992463-5992690 \| ARB_01454 \| NS \| \| JAJVHL010000001.1:5095477-5095728 \| ARB_00837 \| NS \| \| JAJVHL010000001.1:5547587-5547858 \| ARB_04068 \| NS \| \| JAJVHL010000001.1:3376909-3377184 \| ARB_04654 \| NS \| \| JAJVHL010000001.1:4593305-4593584 \| ARB_06997 \| NS \| \| JAJVHL010000001.1:2305307-2305655 \| ND \| NS \| \| JAJVHL010000001.1: 2621464 -2621922 \| ARB_04405 \| NS \| \| JAJVHL010000001.1:3079481-3079980 \| ARB_04550 \| NS \| \| JAJVHL010000002.1:6651538-6651692 \| ND \| NS \| \| JAJVHL010000002.1:1599306-1599481 \| ARB_07303 \| NS \| \| JAJVHL010000002.1:19264-19502 \| ND \| NS \| \| JAJVHL010000002.1:7096770-7097046 \| ND \| NS \| \| JAJVHL010000002.1: 5902910 -5903211 \| ARB_02194 \| NS \| \| JAJVHL010000003.1:1062434-1062648 \| ND \| NS \| \| JAJVHL010000003.1:1010180-1010536 \| ARB_03647 \| NS \| \| JAJVHL010000003.1:1936972-1937471 \| ND \| NS \| \| JAJVHL010000003.1:1937010-1938187 \| ND \| NS \| \| In comparison to *T*. *rubrum* (^‡^), *T*. *interdigitale* (GCA_037576225.1) (**^#^**) and *T*. *benhamiae* RefSeq data (GCF_000151125.1) (^§^)  NS, no similarity; ND, not defined \| \| \| |  |
| --- | --- | --- | --- | --- | --- | --- | --- | --- | --- | --- | --- | --- | --- | --- | --- | --- | --- | --- | --- | --- | --- | --- | --- | --- | --- | --- | --- | --- | --- | --- | --- | --- | --- | --- | --- | --- | --- | --- | --- | --- | --- | --- | --- | --- | --- | --- | --- | --- | --- | --- | --- | --- | --- | --- | --- | --- | --- | --- | --- | --- | --- | --- | --- | --- | --- | --- | --- | --- | --- | --- | --- | --- | --- | --- | --- | --- | --- | --- | --- | --- | --- | --- | --- | --- | --- | --- | --- | --- | --- | --- | --- | --- | --- | --- | --- | --- | --- | --- | --- | --- | --- | --- | --- | --- | --- | --- |


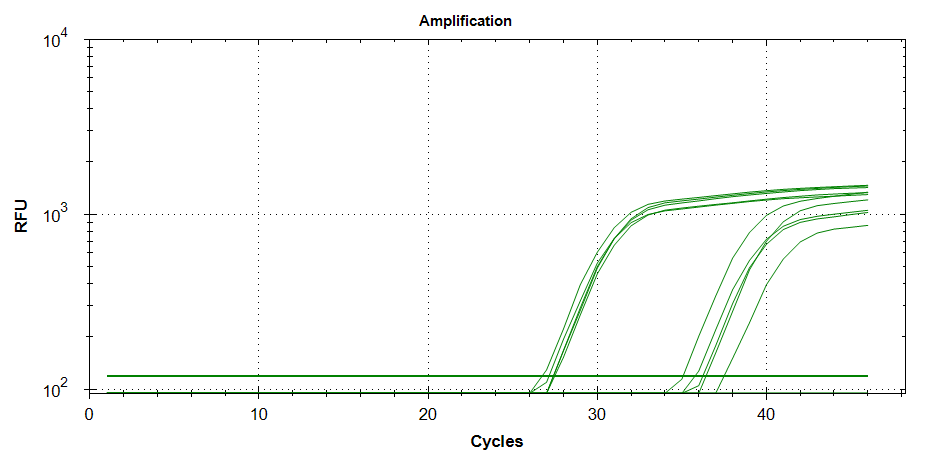


**Fig. S1** Repeatability assay signals from five replicates of each of 1,000 (Ct values between 26 and 30) and 15 (Ct values between 35 and 40) genome copies of *T*. *indotineae* gDNA. The relative standard deviation (RSD) values were 1.5% and 2.5% for 1,000 and 15 copies, respectively
